# Complications Associated with Peripherally Inserted Central Catheters (PICC) in People Undergoing Autologous Hematopoietic Stem Cell Transplantation (HSCT) in Home Hospitalization

**DOI:** 10.3390/ijerph20031704

**Published:** 2023-01-17

**Authors:** Ana María Garcés-Carrasco, Enric Santacatalina-Roig, Carlos Carretero-Márquez, Antonio Martínez-Sabater, Evelin Balaguer-López

**Affiliations:** 1 Oncology and Hematology Department, Hospital Clínico Universitario de Valencia, 46010 Valencia, Spain; 2 Nursing Department, Facultat d’Infermeria i Podologia, Universitat de València, 46010 Valencia, Spain; 3 Nursing Care and Education Research Group (GRIECE), GIUV2019-456, Nursing Department, Universitat de Valencia, 46010 Valencia, Spain; 4 Grupo Asociado de Investigación en Cuidados (INCLIVA), Hospital Clínico Universitario de Valencia, 46010 Valencia, Spain

**Keywords:** catheter-related infections, transplantation, autologous, home nursing, hematology, central catheter access

## Abstract

Introduction: The SARS-CoV-2 pandemic generated the need to keep immunosuppressed patients away from hospital institutions for as long as possible. This in turn stimulated the implementation of a home hospitalization model for autologous hematopoietic stem-cell transplantation (HSCT). Purpose: To analyze whether there are significant differences in post-transplantation complications related to catheters observed in patients treated in the home-transplant care modality compared to patients treated in the hospital. Methodology: Observational, analytical, longitudinal, and retrospective study of cases and controls. A convenience sample was chosen, in which the cases comprised 20 patients included in the home HSCT care model. For each patient, it was considered suitable to propose two controls among those who received autologous transplantation in the last five years with a baseline demographic and pathological profile similar to the case for whom they were control. Results: The home patients achieved an average of 22.4 ± 2.6 days of evolution with an average of 16.4 ± 2.08 days post-transplant, compared to the hospital process with an average of 21.21 ± 4.18 days of evolution and 15.51 ± 3.96 days post-transplant (evolution days *p* = 0.022; post-transplant days *p* = 0.002). A higher percentage of use of parenteral nutrition (*p* = 0.036) and transfusions (*p* = 0.003) was observed during the post-transplant phase in the hospital. The rest of the therapeutic measures did not show significant differences. When analyzing the frequency of adverse effects in the post-transplant phase, a significant increase in neutropenic fever (OR = 8.55) and positive blood cultures (OR = 6.65) was observed in hospital patients. Any other significant differences in other variables related to PICC were found (presence and days of neutropenic fever, catheter infection, complications, pathogens, admission to the ICU, or death). Concerning local complications (pain, DVT, Medical adhesive-related Skin Injury, and erythema), there was more erythema in the hospital (*p* = 0.056). Conclusions: The results obtained indicate that regarding the appearance of complications associated with PICCs in home hospitalization HSCT patients, there are no significant differences compared to hospitalization, so that home care can be a safe context for people with these lines

## 1. Introduction

The improvement in the effectiveness and efficiency of hematopoietic stem-cell transplants (HSCT) has led to an increase in its indications compared to previous decades (when it was only performed on people with advanced or treatment-resistant pathology [1,2]). Some benefits identified for the different pathologies are the main reason for this increase in indications [3]. HSCT consists of administering hematopoietic progenitor cells to a patient from several possible sources (bone marrow, peripheral blood, umbilical cord blood) from different types of donors (autologous, syngeneic, or allogeneic). This process consists of three phases: first phase or conditioning (administration of chemotherapy or radiotherapy to prepare the bone marrow for the receipt of the progenitors and eliminate cancer cells); second phase or infusion or transplant (infusion of the progenitors); third phase or aplasia; and fourth phase or graft phase (in which the progenitors nest in the bone marrow) [1,2,4]. Given the characteristics of the solutions to be infused and according to the different clinical practice guidelines [5,6], these patients usually require a central catheter, with peripherally inserted central catheters standing out in recent years [7,8].

Improvements in treatments and protocols have reduced morbidity and mortality rates [9,10]. So, these treatments and improvements have been allowed to be transferred to the home environment, demonstrating that home care after allogeneic hematopoietic stem-cell transplantation (allo-HSCT) [4] is medically safe and beneficial to the patient [2,11]. These situations of home treatment have been increasing since the outbreak of the SARS-CoV-2 pandemic. In this situation, the initial treatment occurred in the hospital, but the patient was transferred home early for the third phase of the transplant [12].

Given the high use of central vascular access systems, along with the characteristics of the patients, a correct insertion technique is undoubtedly necessary. Moreover, an exhaustive protocolization of the care provided to people with a PICC during the transplant [8,13,14] is also essential for improving success rates and reducing complications and effects on patients’ quality of life [13,15,16,17]. Previous experiences evaluate the effectiveness and safety of home administration of different therapies via PICC, which can be considered an appropriate option [18,19,20]. In addition, training patients and their families about PICC care can benefit patient satisfaction, reduce the incidence of complications, and decrease delays after complications [21].

For this reason, after implementing a home hospitalization model for HSCT, it was proposed to analyze differences in post-transplant complications related to the central catheters observed in patients treated in the home transplant-care modality compared to patients treated in the hospital transplant modality. We hypothesized that there would be no difference in the results regarding the safety of HSCT between patients in conventional hospitalization and those in home hospitalization.

## 2. Materials and Methods

This was an observational, analytical, longitudinal, and retrospective study of matched cases and controls. A convenience sample was considered, in which the total number of patients included in the home HSCT care model from its inception on 20 September 2020, to the completion of the study on 30 November 2021, was considered a case (*n =* 20). Two matched controls (who were not treated in a home-care mode because they were treated before the inception of the same or because they did not have a caregiver) with a similar demographic and pathological profile were chosen for each case. So, the controls were people who had received a hospital HSCT during the last 5 years with similar baseline conditions in terms of diagnosis, comorbidities, stage of the disease, sex, and age to the group of cases (*n =* 40) [22,23]. The HSCT carried out by the Pediatric Oncohematology Service was considered as exclusion criteria. Patients with central vascular accesses different from PICC were also excluded.

Accepting an alpha risk of 0.05 and a beta risk lower than 0.2 in a bilateral contrast, 20 cases, and 40 controls were needed to detect a minimum odds ratio of 6.62. It was assumed that the exposure rate in the control group would be 0.42. A loss of follow-up rate of 0% was estimated by using the Poisson approach.

All patients had a PICC installed in the Vascular Access unit the day before the start of the conditioning phase. Data about several variables were collected: the pre-transplantation baseline, the conditioning phase, transplant and post-transplant treatment, and post-transplant adverse effects, regarding vascular accesses, data on the presence and duration of parenteral nutrition, the number of cures, infections, complications, and catheter withdrawals, among others.

The source of data collection was the review of the clinical record of each patient in the database of the ORION Clinic computer system. An investigator selected the cases and controls following the previously described inclusion criteria. A second investigator conducted data collection for three weeks, and a third investigator was in charge of the audit before data analysis. Given the study’s retrospective nature, the possibility of measurement and selection bias was contemplated [23]. Consequently, an initial collection training session was held to control observation bias. In this training, the definitions of each variable were clarified, and the patient’s record was located. A total of 5% of the cases and controls included were independently reviewed by a second observer to check the proper registration.

Descriptive analysis by groups of the pre-transplant baseline conditions, conditioning phase, transplant phase, and post-transplant treatment was carried out, as well as a description of post-transplant adverse effects. Regarding specific catheter complications (local and systemic), the presence of pain, basal deep vein thrombosis, bruises, bullous lesions of skin, erythema, positive blood culture, catheter infection, and early removal of the catheter were evaluated. To detect significant differences among groups, the Student’s *t*-test was applied for quantitative variables and the Chi-square statistic for qualitative variables, just when parametric application criteria were met. When these parametric application criteria were not fulfilled, the Mann–Whitney U statistic, Kruskal–Wallis for quantitative variables, and Fisher’s “exact F” statistic for qualitative variables were chosen. To check the normality of the variable by groups, the Shapiro–Wilks statistic was applied, and the Levene statistic was applied for homoscedasticity. The Risk Ratio (Odds Ratio) was calculated using logistic regression in those adverse effects where significant differences were detected. In all the analyses, a confidence level of 95% with an alpha of 0.05% was established. For statistical analysis, SPSS 21 (IBM) version 24 was used.

The research received approval from the Ethics and Methodological Committee of the Research Institute in the Health Department.

## 3. Results

A sample of 20 patients whose post-transplantation care was carried out at home and 40 patients whose post-transplantation setting was the hospital (the transplantation area of the Hematology Unit) was analyzed.

No significant differences were found in the baseline conditions of the patients, except for the Karnofsky Scale score (*p* = 0.050), whose score was higher in patients at home (91.7%) than patients in the hospital (87.74%) (Table 1).

Regarding the conditioning phase compared between the home or hospital post-transplant context, significant differences were only found in antibiotic prophylaxis observed in all home patients (a quinolone associated with a cephalosporine or beta-lactam), compared to 72.5% of hospital individuals (sulfonamide or quinolone) (*p* = 0.007). Two antibiotics were associated with 7.5% of hospital patients (sulfonamide with quinolone). In the home setting, two antibiotics were associated with 100% of the individuals (*p* = 0.000). In Table 2, the comparison of average families of antibiotics according to post-transplant context can be observed.

Concerning the transplantation and post-transplantation phases, the home context presented an average of days of evolution of 22.4 ± 2.6, with an average of 16.4 ± 2.08 post-transplant days. At the hospital level, an average evolution time of 21.21 ± 4.18 days was obtained, with an average of 15.51 ± 3.96 post-transplant days (evolution days *p* = 0.022; post-transplant days *p* = 0.002).

In the hospital, a significantly higher percentage of patients who received both parenteral nutrition (*p* = 0.036) and packed red blood cells (*p* = 0.003) was observed during the post-transplant phase. The rest of the registered therapeutic measures did not present significant differences between hospital/home contexts. One patient from each cohort was admitted to the ICU for reasons unrelated to the catheter. Regarding post-transplant antibiotic therapy, it was observed that 7.5% of hospital patients and 5% of home patients were associated with up to five different antibiotics.

When analyzing the frequency of adverse effects observed in the post-transplant phase, a significant increase in the presence of neutropenic fever and positive blood cultures was observed in post-transplant patients in the hospital, with no significant differences in other variables related to central catheters (Table 3).

Regarding the isolated pathogens in blood cultures, up to nine different pathogens were found in the hospital patients (Candida, Staphylococcus Warneri, Staphylococcus Hominis, Coagulase Negative Staphylococcus, Staphylococcus Epidermidis, Streptococcus Mitis, Staphylococcus Aureus, *Escherichia coli*, Serratia Marcenscens) compared to two of them in home patients (Staphylococcus Epidermidis, *Escherichia coli*). Multiresistant pathogens were not isolated from home patients, whereas 12.5% of hospital patients presented this type of pathogen in their blood cultures.

Regarding the complications associated with the catheter at the local level, it was observed that 30% of hospital patients presented with erythema, which only occured in 5% of home patients (*p* = 0.056). Table 3 shows the frequency of local catheter complications in the post-transplant context, highlighting the high presence of erythema and injuries related to adhesives in the puncture area (Medical Adhesive-Related Skin Injury (MARSI)) in the hospital setting. All patients with catheter infections also had erythema or thrombosis. When comparing the average of prophylactic antibiotics and the presence of catheter complications, significant differences were obtained for the group of prophylactic quinolones (without catheter complications 0.87 ± 0.34; with catheter complications 0.59 ± 0.5 (0.37–0.81) (*p* = 0.015).

When analyzing the risk ratio, it was determined that in the hospital it was up to 6.65 times more likely to present with pathogens in blood cultures and to experience neutropenic fever than at home (Table 4).

## 4. Discussion

This study began from an initial sample of 20 post-transplant patients in a home setting who were assigned a total of 40 controls transplanted in a hospital regimen whose baseline conditions did not present differences except for the assessment of the Karnofsky scale. In this sample, the home patients presented a higher score on the scale, exceeding 90%, which implied the ability to perform daily activities without minor signs or symptoms of disease [24,25]. However, hospital patients presented an average score of around 87%, which implies regular activity and the ability to take care of oneself with some sign or symptom of a disease that generates more effort to perform activities [24,25]. The patient’s level of autonomy was a key point for selecting patients in the home HSCT program since they must be in a good general condition to be included in the home protocol. No hospitalized patient had Karnofsky scores lower than 70 (self-care ability unable to perform high-capacity activities [24,25]). Previous studies observed a relationship between a Karnofsky score of less than 70 and the presence of infections, which was also related to the higher durability of the catheter [26].

Concerning the conditioning phase, a higher percentage of antibiotic therapy was observed in the at-home patients because the home program has protocolized pre-transplant prophylactic antibiotic therapy consisting of two antibiotics: a quinolone (levofloxacin) associated with a broad-spectrum antibiotic (ceftriaxone (1 g IV/24 h. in patients with myeloma) or piperacillin-tazobactam (16 g IV/24 h in case of patients with lymphoma); in line with what was recommended by previous experiences and studies [20,24]. In contrast, in the hospital modality, only a broad-spectrum antibiotic was associated with fever spikes in the neutropenia phase (acyclovir and oral levofloxacin +/− oral sulfonamide) according to our usual clinical practice at that time [2,27].

Regarding neutropenia, it was observed that the incidence of patients with neutropenic fever, as well as the average duration of this fever, was lower in the home transplant modality. This result was very significant considering that the neutropenic periods seemed to be significantly longer in the home patients studied, being similar to previous studies that showed that antibiotic prophylaxis with quinolones associated with broad-spectrum antibiotics, in-home transplants were highly satisfactory compared to those performed in the hospital [27], finding a decrease in the days of neutropenic fever observing the same trend as this study.

The use of PICC has played a fundamental and safe role in oncology in recent years [28,29]. Regarding CVCs (central venous catheters), all patients are carriers of a PICC in upper limbs inserted in the hospital vascular access unit. This research showed no significant differences in catheter-related complications and infections between the two modalities. A higher percentage of local complications has been observed in patients who have remained hospitalized. Hospital and home nurses have PICC management protocols and annual training in this care. Home nurses provide training to patients and their caregivers based on hospital protocols. This circumstance may be because both the caregiver and the patient undergoing a home transplant received education about the management of the catheter to prevent possible infections and complications [30,31]. Therefore, this may be one of the reasons why it was believed there are fewer infections in the home setting [32,33]. There is literature that demonstrates a reduction in CVC complications in patients who, both themselves and their caregivers, have received education on catheter maintenance [34,35]. Patients who remained hospitalized did not receive this type of education since nurses act in case of any incident with the CVC. It can also influence the handling time of the catheter. There was no prescribed fluid therapy at home, and the antibiotic was administered through a pump that was changed every 24 or 72 h. Therefore there was less handling of the catheter [6,35,36,37,38]. The influence of differences in the use of antibiotics in the hospital and the home setting and the possible relationship as a factor in the occurrence of complications should be evaluated in the future. However, prophylactic use of antibiotics is not indicated in clinical practice guidelines, even though antimicrobial prevention strategies aimed at these microorganisms could potentially decrease the majority of CVC-related infections. On the other hand, there were differences in the presence of erythema at the insertion point and MARSI, which were more frequent in the hospital setting in this research. It must be taken into account that MARSI are prevalent lesions, although they are usually under-reported [39,40,41].

This study has limitations in the selection, information, and follow-up of the cases derived from the retrospective data collection. In addition, we began from a small sample, which needs to be increased to affirm with more power that the home context is equally safe as the hospital context for post-transplant patients with CVCs. An adequate selection of patients for home transplant (comprehensive assessment, Karnofsky, etc.) allows for maximum safety and minimum complications in managing PICCs, reducing hospital risk factors such as nosocomial bacterial exposure and possible differences in prophylactic management.

## 5. Conclusions

As the conclusion of the study, it can be indicated that home transplantation is a therapeutic option that presents conditions of non-inferiority compared to hospital transplantation. Hospital-based transplants increase the risk of episodes of neutropenic fever and positive blood cultures, with more pathogens isolated in this care setting. Regarding the complications related to the catheter, it is shown that the home regimen can be a safe context for people with these lines

## Figures and Tables

**Table 1:** Baseline conditions of patients undergoing transplantation according to home or hospital post-transplant setting.

Variables	Hospital *n =* 40	Home *n* = 20	*p*
Age	56.4 ± 11.21	56.25 ± 10.14	0.736
Male	60%	60%	1.000
Female	40%	40%	
Comorbidities	37.5%	45%	0.576
Diabetes	10%	15%	0.429
AH	15%	30%	0.152
Dyslipidemia	15%	10%	0.461
KARNOFSKY	87.74 ± 7.62	91.76 ± 3.9	0.050 *
SORROR	1.95 ± 1.45	2.05 ± 1.39	0.808
Myeloma	75%	75%	1.000
Lymphoma	25%	25%	
CR	35.9%	50%	0.297
PR	64.1%	50%	

AH: Arterial Hypertension; CR: complete remission; PR: partial remission * *p* < 0.05.

**Table 2:** Antibiotic Prophylaxis.

Variable	Hospital *n* =40	Home *n* = 20	*p*
Quinolone	0.65 ± 0.48 [0.5–0.8]	1	0.003
Sulfonamide	0.18 ± 0.061 [0.5–0.3]	0	0.048
Cephalosporine	0	0.7 ± 0.1 [0.4–0.9]	0.000
Beta-lactam	0	0.2 ± 0.09 [0.1–0.3]	0.004

**Table 3:** Adverse effects observed in the post-transplant phase.

Variable	Hospital *n =* 40	Home *n =* 20	*p*
Neutropenic fever	87.5%	45%	0.000 *
Days with neutropenic fever	3.28 ± 2.9	0.89 ± 1.6	0.000 *
Days with neutropenia	9.08 ± 1.77	11.5 ± 2.5	0.000 *
Catheter infection Clinical bacteremia (negative culture) *Staphylococcus Aureus* *Candida infection* *Coagulase Negative Staphylococcus* *Staphylococcus Hominis*	10% *0%* *2.5%* *2.5%* *2.5%* *2.5%*	5% *5%* *0%* *0%* *0%* *0%*	0.455
Number of catheter curing	3.7 ± 1.8	4.26 ± 2.13	0.317
Catheter complications *Pain* *Basal Deep Vein Thrombosis (DVT)* *Axillary vein thrombosis* *Bruises* *Bullous lesions of the skin* *Erythema* *Suspected infection*	42.5% *2.5%* *5%* *2.5%* *2.5%* *2.5%* *30%* *2.5%*	25% *0%* *0%* *0%* *10%* *0%* *5%* *10%*	0.149
Number of catheter complications	0.58 ± 0.74	0.25 ± 0.44	0.162
Catheter removal	30%	15%	0.206
Pathogen in BC	42.5%	10%	0.011 *
ICU admission	5%	5%	1.000
Exitus	0%	0%	-

BC: blood culture; ICU: intensive care unit; * *p ≤* 0.05.

**Table 4:** Risk ratio for the presence of adverse effects in the post-transplantation period.

Variable	OR	*p*	CI 95%
Neutropenic fever	8.556	0.001	2.364–30.960
BC pathogen	6.652	0.019	1.357–32.611

OR: Odds Ratio; CI: Confidence Interval; BC: blood culture.

## Data Availability

Data available on request to the corresponding author.
